# PM_2.5_, oxidant defence and cardiorespiratory health: a review

**DOI:** 10.1186/1476-069X-12-40

**Published:** 2013-05-04

**Authors:** Scott A Weichenthal, Krystal Godri-Pollitt, Paul J Villeneuve

**Affiliations:** 1 Health Canada, Air Health Effects Science Division, Ottawa, Canada; 2 University of Toronto, Southern Ontario Centre for Atmospheric Aerosol Research, Toronto, Canada; 3 Health Canada, Population Studies Division, Ottawa, Canada

**Keywords:** Air pollution, Particulate matter, Oxidative stress, Genetic polymorphisms, Anti-oxidants, Cardiovascular health, Respiratory health

## Abstract

Airborne fine particle mass concentrations (PM_2.5_) are used for ambient air quality management worldwide based in part on known cardiorespiratory health effects. While oxidative stress is generally thought to be an important mechanism in determining these effects, relatively few studies have specifically examined how oxidant defence may impact susceptibility to particulate air pollution. Here we review studies that explore the impact of polymorphisms in anti-oxidant related genes or anti-oxidant supplementation on PM_2.5_-induced cardiorespiratory outcomes in an effort to summarize existing evidence related to oxidative stress defence and the health effects of PM_2.5_. Recent studies of PM-oxidative burden were also examined. In total, nine studies were identified and reviewed and existing evidence generally suggests that oxidant defence may modify the impact of PM_2.5_ exposure on various health outcomes, particularly heart rate variability (a measure of autonomic function) which was the most common outcome examined in the studies reviewed. Few studies examined interactions between PM_2.5_ and oxidant defence for respiratory outcomes, and in general studies focused primarily on acute health effects. Therefore, further evaluation of the potential modifying role of oxidant defence in PM_2.5_-induced health effects is required, particularly for chronic outcomes. Similarly, while an exposure metric that captures the ability of PM_2.5_ to cause oxidative stress may offer advantages over traditional mass concentration measurements, little epidemiological evidence is currently available to evaluate the potential benefits of such an approach. Therefore, further evaluation is required to determine how this metric may be incorporated in ambient air quality management.

## Background

Airborne fine particle mass concentrations (PM_2.5_) are used for ambient air quality management worldwide based on years of epidemiological and toxicological evidence suggesting adverse health effects [1,2]. Of the plausible biological mechanisms explaining PM_2.5_ health effects, oxidative stress is often cited as playing an important role in both respiratory and cardiovascular outcomes [3-5]. For this reason, recent attention has focused on PM oxidative burden as an approach to characterizing particle toxicity [6-10] and some findings suggest high spatial variability with proximity to traffic [7,9,10].

However, it is unclear as to how such measures may be incorporated into regulatory air quality management as relatively few studies have specifically examined the modifying role of oxidant defence on PM_2.5-_induced cardiorespiratory morbidity.

Here we review epidemiological evidence related to the role of oxidative stress (and oxidant defence) in PM-induced cardiorespiratory morbidity. In particular, we review studies that explore potential effect modification by polymorphisms in anti-oxidant genes or anti-oxidant supplementation, as well as recent evidence examining the association between PM-oxidative burden and adverse health outcomes. In general, the goal of this review paper was to examine heterogeneity in the magnitude and direction of PM_2.5_ associations according to factors that may impact oxidant defence and in doing so weigh evidence for or against incorporating measures of PM_2.5_ oxidative burden in ambient air quality management. Assays used to evaluate PM oxidative burden are not discussed in detail; a thorough analysis of these methods is available elsewhere [6].

## Methods

Studies were identified through PubMed ( http://www.ncbi.nlm.nih.gov/pubmed/) using the operator AND in combination with search terms including: air pollution/fine particulate matter (PM_2.5_), oxidative stress, oxidative stress genes/polymorphisms, dietary antioxidants, effect modification, respiratory health, and cardiovascular health. For example, typical search terms included: 1) PM_2.5,_ oxidative stress, AND cardiovascular health; 2) PM_2.5,_ oxidative stress, AND respiratory health; 3) PM_2.5_, oxidative potential, AND effect modification; 4) Similar combinations using the terms above. Abstracts of articles retrieved were examined and studies were only included if they were published in English before July 4, 2012 and specifically examined he role of oxidative stress/oxidative stress defence in PM_2.5_-induced cardiorespiratory health effects in humans and/or specifically examined the health effects of PM-oxidative potential.

Citation lists from these articles were also examined. In total, nine studies were identified that met the above criteria and all of these studies were included in the review (Table 1).

**Table 1:** **Studies of genetic polymorphisms, anti-oxidant supplements, and PM**_**2.5**_**-induced cardiorespiratory morbidity**

**Study**	**Design and location**	**Population**	**Polymorphism(s)/treatment**	**Ambient exposure**	**Outcome(s)**^**g**^	**Main findings/effect estimates**
Cardiovascular outcomes						
Schwartz et al. 2005 (11)^a,h^	Normative Aging Study Cohort Boston, US (2000–2004)	497 Caucasian Men Mean Age: 73 years	GSTM1	48-hour average PM_2.5_	HF	Change Per 10 μg/m^3^ GSTM1-present − 3.6% (95% CI: -40.5, 56.2) GSTM1-null − 34% (95% CI: -53, -7.2)
Chahine et al. 2007 (13)^a,h^	Normative Aging Study Cohort Boston, USA (2000–2005)	539 Caucasian Men Mean Age: 73 years	GSTM1 HMOX-1 promoter	48-hour average PM_2.5_	SDNN, HF, LF	Change per 10 μg/m^3^ GSTM1-present SDNN: -2.0% (95% CI: -11.3, 8.3) HF: -4.0% (95% CI: -24.8, 22.6) LF:-0.6% (95% CI: -19.0, 22.0) GSTM1-null SDNN: -10.5% (95% CI: -18.2, -2.2) HF: -24.2% (95% CI: -39.2, -5.5) LF: -17.0% (95% CI: -31.0, -0.2) HMOX-1 short repeat SDNN: 7.4% (95% CI:-8.7, 26.2) HF: 8.9% (95% CI:-27.1, 62.8) LF: 14.0% (95% CI: -18.6, 59.5) HMOX-1 long repeat SDNN: -8.5% (95% CI: -14.8, -1.8) HF: -20.1% (95% CI: -32.9, -5.0) LF: -14.0% (95% CI: -25.7, -0.5) GSTM1-null and HMOX-1 long repeat SDNN: -12.7% (95% CI: -20.6, -3.9) HF: -27.8% (95% CI: -43.0, -8.5) LF: -20.1% (95% CI: -34.5, -2.7)
Park et al. 2006 (12)^b,h^	Prospective Cohort Boston, US (2000–2004)	518 Caucasian Men Mean Age: 73 years	HFE	48-hour average PM_2.5_	HF LF LF/HF SDNN	Change Per 10 μg/m^3^ HFE wild type HF: -31.7% (95% CI: -48.1, -10) The authors noted that similar associations were observed for SDNN, LF, and LF/HF but the data was not shown.
Ren et al. 2010 (15)^c,h^	Prospective Cohort Boston, US (1995–2006)	1000 Caucasian Men Mean Age: 72 years	HFE NQO1 CAT GSTM1 GSTP1 GSTT1 HMOX-1	7-day PM_2.5_ moving average	Total plasma homocysteine	Change Per 4.56 μg/m^3^ HFE wild type 1.81% (95% CI: 0.46, 3.16) HFE-variant (rs1800562) − 2.5% (95% CI: -5.68, 0.68) CAT wild type 0.75% (95% CI: -0.85, 2.35) CAT-variant (rs2300181) 2.84% (95% CI:’.06, 4.62)
Madrigano et al. 2010 (14)^d,h^	Prospective Cohort Boston USA (1998–2008)	809 Caucasian Men Mean Age: 74 years	GSTM1 HMOX-1 HFE	1-3 day PM_2.5_ moving average	sICAM-1 sVCAM-1	Polymorphisms in GSTM1, HMOX-1, or HFE genes did not modify the relationship between PM_2.5_ exposure and serum concentrations of sICAM-1 or sVCAM-1 (effect estimates not reported)
Romieu et al. 2005 (31)^e^	Randomized Double-Blind Trial	60 Adults Mean Age: 80 years	2 g/day fish oil or 2 g/day soy oil	Daily Indoor PM_2.5_	HF LF pNN50 SDNN rMSSD	Change Per 8 μg/m^3^ Pre-Supplementation Group (fish oil) Log10 HF: -54% (95% CI: -72, -24) Log10 LF: -48% (95% CI :-69, -15) Log10 pNN50: -44% (95% CI : -56, -27) Log10 SDNN : -27% (95% CI : -37, -16) Log10 rMSSD: -32% (95% CI: -43, -19) Supplementation Phase (fish oil) Log10 HF: -7% (95% CI: -20, 7) Log10 LF: -10% (95% CI : -22, 3) Log10 pNN50: -5% (95% CI: -12, 2) Log10 SDNN: -0.5% (95% CI: -4, 3) Log10 rMSSD: 0.02% (95% CI: -7, 8)
Respiratory Outcomes						
Breton et al. 2011 (30)^f^	Prospective Cohort California, USA (1993–2004)	2106 Children Mean Age (baseline): 10 years	GSS	Yearly Average PM_2.5_	Lung function growth in: FEV_1_ FVC MMEF	Per 22.2 μg/m^3^ GSS-H0100000 FEV_1_: -124 ml (95% CI: -203, -45.3) FVC: -92.9 ml (95% CI: -186, 0.2) MMEF: -193.9 ml/s (95% CI: -352.2, -35.6) Other GSS Haplotypes FEV_1_: -49 ml (95% CI: -182, 83.9) FVC: -106.8 ml (95% CI: -247, 33.2) MMEF: -70.9 ml/s (95% CI: -309, 167.3)

^a^ Adjusted for age, smoking, body mass index, diastolic blood pressure, fasting blood glucose, alcohol consumption, cardiovascular medications, season, and temperature.

^b^ Adjusted for age, smoking, body mass index, mean arterial blood pressure, high-density lipoprotein cholesterol, history of ischemic heart disease, fasting blood glucose, alcohol consumption, cardiovascular medications, season, day of the week, and temperature.

^c^ Adjusted for age, body mass index, systolic blood pressure, smoking, alcohol consumption, serum creatinine, plasma folate, and vitamins B6 and B12.

^d^ Adjusted for age, temperature, obesity, smoking, statin use, and diabetes.

^e^ Adjusted for age, sex, heart rate, hypertension, body mass index, and time of day.

^f^ Adjusted for height, sex, body mass index, asthma diagnosis, respiratory illness at testing, exercise, smoking, ethnicity, cohort, town, field technician, GSTM1, and ancestry.

^g^ HF, high frequency heart rate variability; LF, low frequency heart rate variability; SDNN, standard deviation of normal to normal intervals; pNN50, percentage of normal RR intervals differing by more than 50 ms; rMSSD, root mean square of the sum of square differences between adjacent intervals; ICAM-1, soluble intercellular adhesion molecule; sVCAM-1, soluble vascular cell adhesion molecule; NQO1, NAD(P)H dehydrogenase [quinine] 1; CAT, catalase; GSH, reduced glutathione; GSS, GSH synthetase; FEV_1_, forced expiratory volume in 1-second; FVC, forced vital capacity; MMEF, maximal mid-expiratory flow.

^h^ Previously reviewed by Zanobetti et al. [21].

In particular, studies listed in Table 1 include 4 new studies [11-15] as well five studies [16-20] previously discussed by Zanobetti et al. [21].

## Results

### Polymorphisms in anti-oxidant related genes

#### Cardiovascular health effects

Reactive oxygen species (ROS) are produced in the body both through natural biological processes as well as in response to external stimuli such as air pollution [3,5]. As such, mechanisms have evolved to maintain cellular redox equilibrium to counter the potential adverse health effects of oxidative stress including damage to cellular macromolecules (e.g. proteins, DNA, membranes), inflammation, and cytotoxicity [3,5]. A number of anti-oxidant related genes have been identified and several studies have examined the degree to which polymorphisms in these genes may modify responses to PM with an aerodynamic diameter less than 2.5 μm (PM_2.5_) (Table 1).

Zanobetti et al. [21] recently reviewed a number of studies examining gene-air pollution interactions with respect to cardiovascular morbidity. Of the sixteen studies reviewed, five studies specifically examined the effects of PM_2.5_ exposure according to gene polymorphisms related to anti-oxidant defence [16-20]. However, all five of these studies were conducted in the same population of elderly Caucasian men (The Normative Aging Study) [22]. Polymorphisms in genes coding for glutathione S-transferase enzymes (GSTM1, GSTP1, GSTT1) were examined most often but variants in genes for heme oxygenase-1 (HMOX-1), hemocromatosis (HFE), NAD(P)H dehydrogenase [quinine] 1 (NQO1), and catalase (CAT) were also examined.

The GST family of enzymes play a crucial role in anti-oxidant defence as they catalyze the conjunction of ROS with reduced glutathione (GSH), thereby neutralizing the source of oxidative stress [23,24]. Polymorphisms in GST genes are common, and studies reviewed by Zanobetti et al. [21] suggest that these polymorphisms may impact susceptibility to PM_2.5_-induced changes in autonomic control of the heart. Specifically, elderly male subjects with the GSTM1-null genotype (i.e. non-functional genotype) displayed decreased high-frequency heart rate variability (HRV) with increased ambient PM_2.5_ but this association was not observed among subjects with the functional allele [16]. Interestingly, this study also reported that statin use (a lipid lowering drug with anti-oxidant activity) among GSTM1-null subjects eliminated the association between PM_2.5_ and HRV. In an extended follow-up of the same population, Chahine et al. [18] reported similar findings with respect to GSTM1, HRV, and PM_2.5_ and also reported that polymorphisms in the HMOX-1 promoter modified the effect of PM_2.5_ on HRV. However, tests for interaction between PM_2.5_ and gene polymorphisms were not statistically significant for GSTM1 (0.12 ≤ p ≤ 0.15) or HMOX-1 (0.06 ≤ p ≤ 0.11). HMOX-1 is an inducible enzyme that is produced as part of cellular oxidative stress defence [25], and Chahine et al. [18] reported that ambient PM_2.5_ was inversely associated with HRV among subjects with a high number of GT repeats in the HMOX-1 promoter region (a gene variant known to decrease HMOX-1 expression) but not among subjects with short repeats. In addition, statistically significant (0.008 ≤ p ≤ 0.04) three-way interactions were reported between PM_2.5,_ HMOX-1, and GSTM1 with the strongest inverse associations between PM_2.5_ and HRV observed among subjects who had both the GSTM1 null genotype and long GT repeats in the HMOX-1 promoter. In a more recent study, Madrigano et al. [19] examined the potential impact of anti-oxidant gene polymorphisms (GSTM1, HMOX-1, HFE) on the relationship between PM_2.5_ and serum concentrations of soluble adhesion molecules involved in endothelial dysfunction (soluble intercellular adhesion molecule, sICAM-1; soluble vascular cell adhesion molecule, sVCAM-1). Ambient PM_2.5_ concentrations were not associated with changes in sICAM-1 or sVCAM-1 and evidence of effect modification was not observed for these outcomes (interaction p-value not reported).

Park et al. [17] also examined gene-environmental interactions with respect to ambient PM_2.5_ and HRV but focused on polymorphisms in the HFE gene. The protein product of the HFE gene participates in iron uptake into cells, thereby reducing its ability to participate in Fenton reactions that generate reactive oxygen species [3,26]. Two functional polymorphisms in the HFE gene are known to increase iron uptake and Park et al. [17] reported that ambient PM_2.5_ was inversely associated with high-frequency HRV among subjects with the wild type HFE gene but not among those with either of the two gene variants (interaction p-value = 0.02). Moreover, Ren et al. [20] reported that HFE gene variants also modified the association between ambient PM_2.5_ and total plasma homocysteine levels with a positive association observed among carries of the wild-type HFE gene and an inverse non-statistically significant association observed for carriers of the variant gene (interaction p-value < 0.05). Total plasma homocysteine levels are a maker of systemic oxidative stress [27] and are independent predictors of cardiovascular morbidity [28,29]. In addition, Ren et al. [20] also reported evidence of effect modification for a polymorphism in the catalase (CAT-rs2300181) gene whereby PM_2.5_ was positively associated with plasma homocysteine in carriers of the variant gene but not in carriers of the wild-type gene (interaction p-value < 0.05). Catalase is an anti-oxidant enzyme that converts hydrogen peroxide to water and oxygen and polymorphisms in this gene are thought to decrease gene transcription and thus decrease anti-oxidant capacity [30].

#### Respiratory health effects

Minelli et al. [31] recently reviewed a number of studies examining interactions between anti-oxidant genes and air pollution exposures with respect to respiratory function and airway disease. However, of the seventeen studies reviewed, only three included measures of ambient PM_2.5_[32-34] and two of these studies [32,34] did not examine the impact of PM_2.5_ exposure on respiratory effects according to genotype; instead, genotype was treated as a covariate in the analysis. Specifically, Hong et al. [32] examined the relationship between ambient PM_2.5_ and lung function in a panel of Korean school children and reported that a 1-day lag in PM_2.5_ levels were associated with decreased daily mean peak expiratory flow rate (−0.54 L/min per 1 μg/m^3^, p < 0.01); adjusting for polymorphisms in GSTM1 or GSTT1 genes (null versus present) did not substantially change this association. Islam et al. [34] examined asthma incidence among school children in southern California and reported increased risk among carriers of the GSTM1-null allele (Hazard Ratio (HR): 1.61, 95% CI: 1.2, 2.2) and decreased asthma risk among carriers of the Valine^105^ allele for GSTT1 (HR: 0.60, 95% CI: 0.4, 0.8). A second polymorphism in the GSTT1 gene that decreases anti-oxidant activity was also associated with increased asthma risk (HR: 1.40, 95% CI: 1.1, 1.9); adjusting for ambient PM_2.5_ did not change effect estimates for gene variants but associations for PM_2.5_ were not reported. Finally, the third study did not present data for PM_2.5_ but only mentioned that significant interactions were not observed between non-ozone pollutants (including PM_2.5_) and polymorphisms in the HMOX-1 gene with respect to asthma incidence during adolescence [33].

One additional study was identified that examined gene-environment interactions with respect to PM_2.5_ and respiratory morbidity since the review by Minelli et al. [31]. Specifically, Breton et al. [11] examined whether polymorphisms in genes involved in glutathione (GSH) synthesis modify the impact of PM_2.5_ exposure on lung function growth in children (i.e. the increase in lung function over time). In particular, a polymorphism in glutathione synthetase (GSS) (the enzyme that catalyses the production of reduced glutathione required to respond to oxidative stress) was independently associated with decreased lung function growth among children. In addition, PM_2.5_ exposure was associated with greater decreases in lung function growth among children with the GSS-H0100000 variant than those with other variants (Table 1); however, tests for interaction were not statistically significant (0.44 ≤ p ≤ 0.70).

### Anti-oxidant supplementation

Few studies have specifically examined the impact of anti-oxidant supplementation on the cardiorespiratory effects of PM_2.5_ exposure. Romieu et al. [12] examined the impact of omega-3 polyunsaturated fatty acid supplementation on the relationship between indoor PM_2.5_ and HRV among elderly nursing home residents in Mexico City. In this study, subjects were randomized to receive treatment with either fish oil (2 g/day) or soy oil (2 g/day) and were followed for six months (1-month before supplementation and 5-months after). Prior to supplementation, a 1-standard deviation change in indoor PM_2.5_ (8 μg/m^3^) was associated with statistically significant decreases in time and frequency domain measures of HRV in both groups. After supplementation, both supplements blunted the effect of same-day indoor PM_2.5_ on HRV but this effect was greatest for fish oil which contained a higher concentration of omega-3 fatty acids (Table 1). Tong et al. [13] reported similar findings with respect to fish oil supplementation and HRV among elderly adults in a more recent study of controlled exposure to concentrated ambient particles but they did not present findings for PM_2.5_ specifically.

### PM oxidative stress potential

Studies examining the potential health effects of ambient PM_2.5_ oxidative burden were not identified, but two recent studies examined the health impacts of ambient PM_10_ oxidative burden (Table 2). Specifically, Tonne et al. [14] examined the relationship between ambient PM_10_ oxidative burden and carotid intima-media thickness, a measure of subclinical atherosclerosis. Most participants were Caucasian men and PM_10_ and PM_10_ oxidative burden were estimated at the center of each participant’s postal code using geostatistical models. The primary exposure variable was PM_10_ weighted by oxidative burden averaged during the 52 weeks prior to assessment of intima-media thickness. This study measured PM oxidative burden as the ability of PM extracts to deplete antioxidants from a simulated respiratory tract lining fluid model (RTLF). This chemical model included a composite solution of the three major water soluble antioxidants (glutathione, urate, and ascorbate) at physiological concentrations which serve as the first line of protective defence in the airway against the oxidative activity of PM [35]. This assay measures the intrinsic oxidative potential of PM and does not capture oxidative stress caused by cellular activation; therefore, it underestimates the total oxidative burden of PM. In this study, an interquartile change in PM_10_ weighted by oxidative burden was associated with a 1.2% (95% CI: 0.2, 2.2) increase in intima-media thickness; however, a stronger relationship was observed for PM_10_ mass concentration measurements (5% increase, 95% CI: 1.9, 8.3).

**Table 2:** Studies of PM oxidative potential and cardiorespiratory morbidity

**Study**	**Design and location**	**Population**	**Ambient exposure**	**Outcome(s)**^**c**^	**Main Findings/effect Estimates**
Tonne et al. 2012 (33)^a^	Prospective Cohort, London, United Kingdom (2002–2004)	2347 Adults Mean Age: 61 years	Predicted weekly average PM_10_ and PM_10_ Oxidative Potential (PM_10_*OP) at the center of each participants’ postal code of residence using a geostatistical spatial-temporal model	Carotid Intima-Media Thickness	Change Per 1.5/m^3^ PM_10_*OP 1.2% (95% CI: 0.2, 2.2)
Strak et al. 2012 (34)^b^	Panel Study (Repeated Measures) Utrecht, Netherlands	31 Adults Mean Age: 22 years	5-hour exposure to PM_10_ oxidative potential at five different locations	FE_NO_ FVC	Effect estimates for FE_NO_ ranged from −0.45% to 0.11% per 38.71 change in PM_10_*OP depending on the co-pollutant included in the model
					Effect estimate for FVC ranged from −0.01% to 0.05% per 38.71 change in PM_10_*OP depending on the co-pollutant included in the model

^a^ Adjusted for age, sex, smoking, BMI, season, and year. ^b^ Adjusted for temperature, relative humidity, season, pollen counts, respiratory infection, and co-pollutants. ^c^ FE_NO_, Exhaled NO; FVC, forced vital capacity.

Strak et al. [15] used the same assay to evaluate the acute respiratory health effects of PM_10_ oxidative burden in a panel of healthy adult volunteers. Subjects were exposed to ambient air pollution at five different locations including two traffic sites, an urban background location, an underground train station, and a farm, and detailed air pollution measurements were collected during 5-hour exposures at each site. In this study, PM_2.5_, PM_10_, and PM_10_ oxidative burden (measured as the ability of filter extracts to deplete reduced glutathione and ascorbate) did not have an important impact on acute changes in exhaled NO or lung function; however, significant relationships were reported for ultrafine particles and NO_2_/NOx.

## Discussion

In total, seven studies were identified that specifically examined the impact of polymorphisms in anti-oxidant genes or anti-oxidant supplementation on PM_2.5_ induced cardiorespiratory morbidity. Two additional studies were identified that examined the impact of PM_10_ oxidative burden on cardiorespiratory morbidity; however, studies of PM_2.5_ oxidative burden were not identified. In general, studies to date have primarily examined the impact of polymorphisms in anti-oxidant genes, anti-oxidant supplementation, or PM oxidative potential on the acute cardiorespiratory health effects of PM_2.5_ exposure (48 hours to 7 days) with only one study [11] examining effect modification over longer time-periods.

Of the six studies that examined the impact of anti-oxidant gene polymorphisms on PM_2.5_ induced cardiorespiratory morbidity, three examined heart rate variability as the primary outcome, one examined serum concentrations of soluble adhesion molecules involved in endothelial dysfunction, and one examined total plasma homocysteine. In general, stronger inverse associations were observed between PM_2.5_ and HRV among subjects with genetic polymorphisms that impaired oxidant defence; however, all of these studies were conducted in the same population of elderly Caucasian men and it is not clear if these findings are generalizable to other populations. Nevertheless, a recent meta-analysis generally supports an inverse relationship between PM_2.5_ and HRV suggesting worse cardiovascular prognosis with increased exposure [36]. It is difficult to gage the consistency of other findings as evidence is limited to single studies for the remaining outcomes. Nevertheless, findings from a double-blind trial of dietary anti-oxidant supplementation support the role of oxidative stress in PM_2.5_-induced changes in HRV as anti-oxidant supplementation was found to reduce inverse associations between PM_2.5_ and both time and frequency-domain measures of HRV [12]. In addition, a polymorphism in the enzyme needed to produce reduced glutathione (important for oxidant defence) was found to modify the impact of PM_2.5_ exposure on lung function growth among children, although tests for interaction were not statistically significant [11]. Therefore, although the number of studies is limited, existing evidence is consistent with a potential modifying role of oxidant defence in PM_2.5_ induced cardiorespiratory morbidity. As a result, an exposure measure that captures the ability of PM_2.5_ to cause oxidative stress may offer advantages over traditional mass concentration measurements. However, as outlined below, sufficient evidence is not yet available to determine the precise form that such a metric may take.

Conceptually, PM oxidative burden is an appealing exposure metric for epidemiological analysis as it aims to capture the ability of PM to modify a biological process known to contribute to adverse cardiorespiratory health effects. However, identifying the most important predictors of this measure may not be a straightforward process as some authors have reported heterogeneous associations between PM_2.5_ components and oxidative capacity [37]. While the studies reviewed suggest that oxidant defence may impact susceptibility to PM-induced cardiorespiratory morbidity, few epidemiological studies have specifically examined PM oxidative burden. As a result, little evidence is available to gauge the potential role of this metric in ambient air quality management. Specifically, studies of PM_2.5_ oxidative burden were not identified and only two studies examined the association between PM_10_ oxidative burden and cardiorespiratory morbidity; neither reported strong associations. However, the first of these studies [14] relied on statistical models to estimate PM_10_ oxidative burden and the authors reported that the performance of this model was lower (R^2^ = 0.47) than the model for PM_10_ (R^2^ = 0.83) in cross validation studies. Therefore, increased exposure measurement error may have attenuated effect estimates for the association between PM_10_ oxidative burden and carotid intima-media thickness to a larger extent than those for PM_10_ mass concentration. Nevertheless, the second study monitored PM_10_ oxidative potential exposures directly but did not observe a meaningful association between this measure and acute changes in exhaled NO or lung function in healthy adults [15]. As a number of methods are available to evaluate PM oxidative burden [6], one explanation for the null findings above may be that the particular assay used did not adequately reflect the true “biological” oxidative potential of the particles monitored. Indeed, the method employed in these studies relies on an acellular assay of simulated respiratory tract lining fluid containing antioxidants known to be present in the lung: glutathione and ascorbate [6]. As the authors acknowledge, this assay does not reflect total oxidative burden which requires cell/tissue interactions with particles, only the inherent ability of particles to deplete antioxidants present in the respiratory tract lining fluid. Nevertheless, oxidative potential measures from this assay have been shown to vary with proximity to important sources of air pollution such as traffic [9,10] and further evaluation of an expanded set of health outcomes with both acute and chronic exposure intervals are required before the validity of this measure can be fairly assessed. Likewise, other measures of PM oxidative burden should also be explored in future epidemiological analysis of air pollution health effects in order to identify those that most reliably predict integrated particle toxicity and consider multiple determinants (e.g. transition metals, polycyclic aromatic hydrocarbons) of oxidant capacity. Indeed, if identified such measures may offer additional methods of communicating regional differences in air quality and potentially move us beyond the assumption that PM mass concentrations pose equal health risks regardless of sources and/or composition in a given area.

## Conclusions

In general, existing evidence suggests that altered oxidant defence may have a meaningful impact on PM_2.5_ health effects, particularly for HRV (a measure of autonomic function) which was the most common outcome examined in the studies reviewed. Nevertheless, relatively few studies have specifically examined the impact of oxidant defence on associations between ambient PM_2.5_ and cardiorespiratory morbidity and little is known about the association between PM-oxidative potential and adverse health outcomes. In addition, evidence to date is largely limited to a population of elderly Caucasian men and further evaluation of effect modification by polymorphisms in anti-oxidant genes or dietary anti-oxidants is warranted as these factors may play an important role in determining population susceptibilities to PM-induced health effects. Moving forward, further effort is required in evaluating how PM-oxidative burden may be incorporated in ambient air quality management. This will include evaluation of multiple metrics as well as their use in epidemiological studies of both the chronic and acute health effects of particulate air pollution. If identified, a reliable and valid measure of integrated particle toxicity would offer an additional means of communicating regional differences in air quality and may allow regulators to directly target specific determinants of particle toxicity.

## Abbreviations

CAT: Catalase; CI: Confidence interval; GSS: Glutathione synthetase; GST: Glutathione S-transferase; HF: High frequency; HFE: Hemocromatosis; HMOX-1: Heme oxygenase-1; HR: Hazard ratio; HRV: Heart rate variability; LF: Low frequency; NAS: Normative Aging Study; NCQO1: NAD(P)H dehydrogenase [quinine] 1; OP: Oxidative potential; pNN50: Percentage of normal RR intervals differing by more than 50 ms; PM: Particulate matter; PM2.5: Fine particulate matter (aerodynamic diameter less than 2.5 μm); PM10: Particulate matter with aerodynamic diameter less than 10 μm; rMSSD: Root mean square of the sum of square differences between adjacent intervals; SDNN: Standard deviation of normal to normal intervals; sICAM-1: Soluble intercellular adhesion molecule; sVCAM-1: Soluble vascular adhesion molecule.

## Competing interests

The authors declare that they have no competing interests.

## Authors’ contributions

SW was the lead author of the manuscript. KGP and PV provided many helpful improvements to the first draft of the paper and assisted in writing the final version. All authors read and approved the final manuscript.
